# Malignant Pilomatricoma with Lung Metastases: A Case Report and Literature Review

**DOI:** 10.1055/s-0044-1792022

**Published:** 2024-12-24

**Authors:** Nae-Ho Lee, Jung Kyun Park, Si-Gyun Roh, Jin Yong Shin, Yoon Kyu Chung, Kyu Yun Jang

**Affiliations:** 1 Department of Plastic and Reconstructive Surgery, Medical School of Jeonbuk National University, Jeonju, Republic of Korea; 2 Research Institute of Clinical Medicine of Jeonbuk National University-Biomedical Research Institute of Jeonbuk National University Hospital, Jeonju, Republic of Korea; 3 Department of Pathology, Medical School of Jeonbuk National University, Jeonju, Republic of Korea

**Keywords:** malignant pilomatricoma, lung metastasis, case reports

## Abstract

Malignant pilomatricoma, an extremely rare tumor arising from hair follicles, most commonly occurs on the head, neck, and back. This tumor exhibits several noteworthy characteristics. First, it frequently recurs if it is incompletely excised and can occasionally metastasize to the lungs, bones, and lymph nodes. Additionally, it possesses unique histological features that aid in differentiating it from its benign counterpart, including atypical cells, multiple mitoses with ghost cells, nuclear polymorphisms, and necrosis accompanied by serial desmoplasia. While no definitive criteria have been established for the surgical management of this malignant tumor, it is recommended to perform wide local excision with a safety margin of at least ≥5 mm. To the best of our knowledge, very few cases of malignant pilomatricoma with lung metastasis have been reported in Korea. Here we report the case of a patient diagnosed of malignant pilomatricoma with lung metastasis who underwent wide local excision for a lesion on the flank.

## Introduction

Malignant pilomatricoma is a rare malignant tumor that originates from hair follicles and is infrequently encountered in clinical practice. Due to its overlapping histological features with benign pilomatricoma, it can be easily misdiagnosed, leading to a delayed diagnosis. When treating a patient with malignant pilomatricoma, the plastic surgeon must plan for surgery while taking into account the tumor's aggressiveness and potential for distant metastasis. It is important to recognize this entity, as malignant pilomatricoma can recur if not completely excised and may metastasize to the lungs, bones, and lymph nodes. We report our experience with a patient diagnosed with malignant pilomatricoma with lung metastasis who underwent wide local excision for a lesion on the flank. The patient provided written informed consent for the publication of this report and all accompanying images. 1 2

## Case

**Fig. 1:**
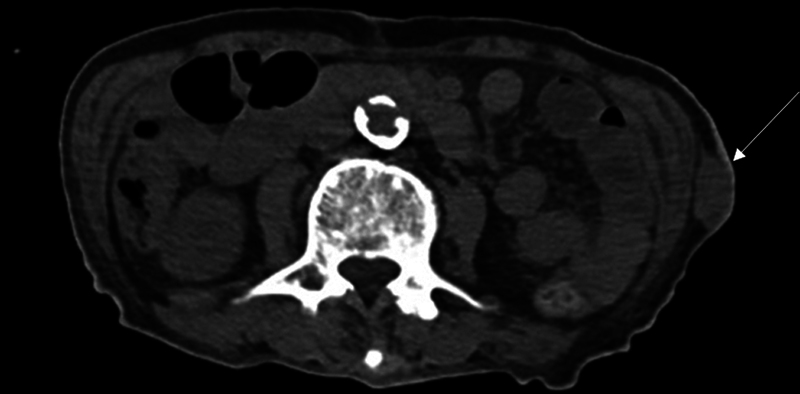
Computed tomography scans revealed a huge mass measuring 4.5 × 2.2 cm in the flank, with reports suggesting possible malignancy.

**Fig. 2:**
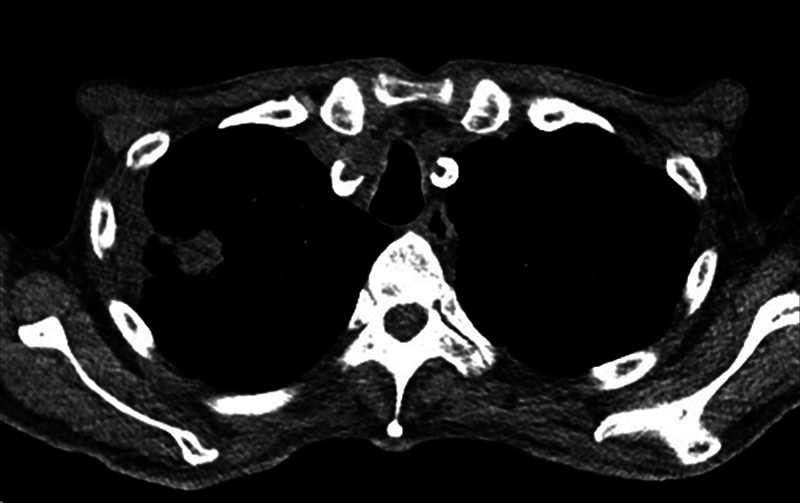
Computed tomography scans revealed a spiculated mass with infected bullae measuring 3.3 cm in the right upper lobe, with reports suggesting possible malignancy.

**Fig. 3:**
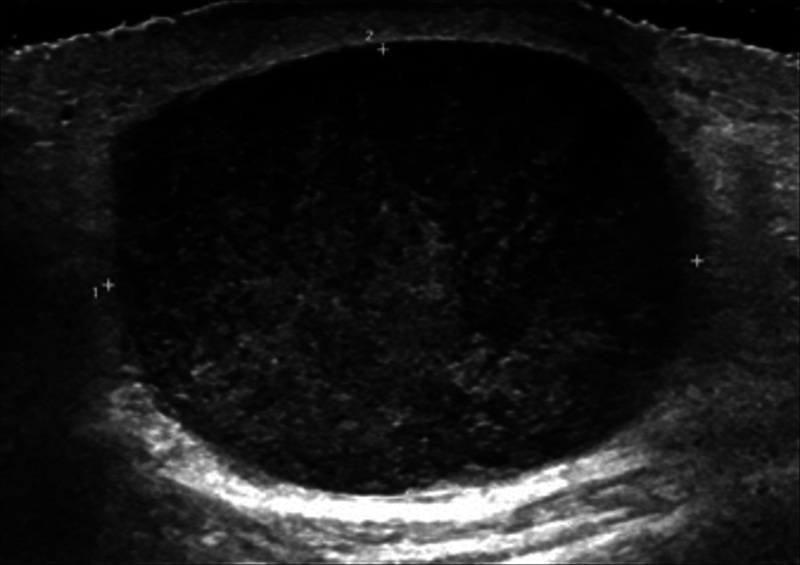
Ultrasound revealed a heterogeneously hypoechoic subcutaneous mass lesion with prominently increased vascularity and perilesional soft tissue edematous changes.

An 84-year-old man presented to our clinic with a palpable mass on his left flank. The mass was quite large and protruding, and no other cutaneous symptoms were observed. The patient did not remember exactly when the lump had been present, but he said it had been palpable for months, and there was a significant weight loss at the time. The mass was large enough to be clearly visible to the naked eye, had no tenderness, and showed no clinical symptoms other than weight loss. Prior to his visit, computed tomography (CT) scans and ultrasonography were performed at other hospitals. The CT scans revealed a huge mass measuring 4.5 × 2.2 cm in the flank, with reports suggesting possible malignancy ( Fig. 1 ). CT also showed a spiculated mass in the right upper lobe, which was suspicious for lung cancer ( Fig. 2 ). Ultrasonography showed a heterogeneously hypoechoic subcutaneous mass lesion with prominently increased vascularity and perilesional soft tissue edematous changes ( Fig. 3 ).

**Fig. 4:**
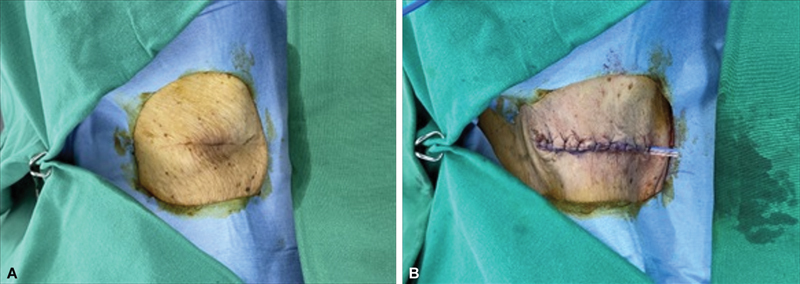
( **A** ) A large protruding lump was identified. ( **B** ) Postoperative view after wide local excision.

Upon initial examination, a large protruding lump was identified on the patient's flank ( Fig. 4A ). Consequently, wide local excision of the mass on the left flank and a local advancement flap under local anesthesia were planned. The proposed incision line was marked with gentian violet solution around the mass on the left flank. Using a no. 15 surgical blade, the mass was excised with 10-mm safety margin along the proposed incision line, revealing multiple pieces of grayish-brown tissue. The excised specimen was sent to our pathology department for frozen biopsy, the results of which indicated that all margins were negative for malignancy. Since the skin flap could be moved directly toward the defect, the flap was elevated and advanced ( Fig. 4B ).

This patient was initially suspected to have lung cancer, and we were requested to perform surgery for a cutaneous metastatic mass. After wide local excision, however, malignant pilomatricoma was diagnosed based on the biopsy results. Furthermore, this cutaneous mass was confirmed to be the primary cancer from which the lung metastases originated.

**Fig. 5:**
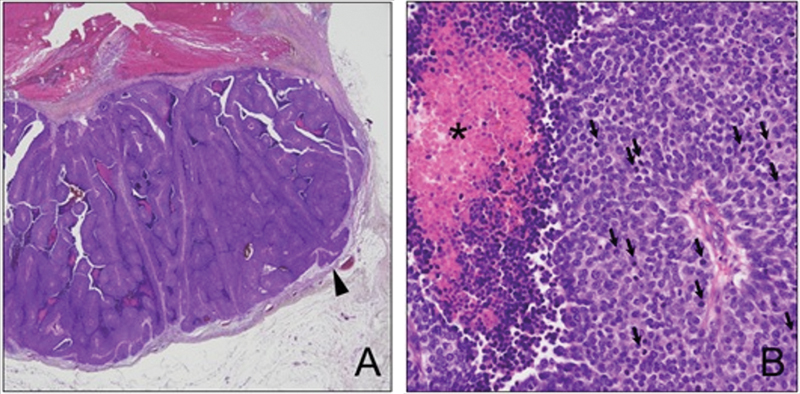
Histological findings of malignant pilomatricoma. ( **A** ) At low magnification, the tumor displayed hyperchromatic basaloid cells and infiltrates on the periphery ( *arrowhead* ). ( **B** ) In a high-power image, the basaloid tumor cells exhibited significant cytologic atypia and frequent mitosis (indicated by *arrows* ) along with ghost cells (denoted by an *asterisk* ). Original magnification: ( **A** ) ×20; ( **B** ) ×400.

Permanent histopathology revealed malignant pilomatricoma with hair follicular differentiation as a malignant skin adnexal tumor. The tumor was composed of hyperchromatic basaloid cells and infiltrates on the periphery ( Fig. 5A ). The pathologic findings also included basaloid tumor cells exhibiting significant cytologic atypia and frequent mitosis with ghost cells, consistent with malignant pilomatricoma ( Fig. 5B ). Immunohistochemistry was not only strongly positive for creatine kinase and epithelial membrane antigen but also focally positive for thyroid transcription factor-1, synaptophysin, CD56, and chromogranin. Further evaluations, including a bone scan, whole-body positron emission tomography/CT, and chest CT, were performed to rule out additional distant metastasis, and no further metastasis was identified.

To treat the lung metastasis, a video-assisted thoracoscopic biopsy of the lung lesion was done, which confirmed metastasis from the primary cancer on the flank. Several courses of chemotherapy with intravenous cisplatin and 5-fluorouracil were given. Follow-up CT after each cycle showed shrinkage of the lung metastasis. During the follow-up period, the patient regularly visited our clinic and experienced no issues.

After surgery, the patient recovered well without any complications. At the 6-week follow-up, the patient carried on with daily life without difficulties and was able to receive medical treatment for lung metastasis.

## Discussion

Malignant pilomatricoma originates from hair follicles and is very rare in clinical practice. It presents as an asymptomatic, potentially ulcerative, enveloped subcutaneous nodule or cystic mass, and it cannot be reliably distinguished from benign adnexal tumors based on clinical appearance alone. 3 4 5 6

Malignant pilomatricomas are primarily observed in elderly individuals between their sixth and seventh decades of life, with a male-to-female ratio of 5:1. 7 These tumors most commonly occur on the head, neck, and back, ranging in size from 1 to 10 cm, and are typically located in the deep dermis. 2 4 In the present case, the mass was discovered on the patient's flank as a visibly protruding lump that could be seen with the naked eye.

In some instances, malignant pilomatricoma has been misdiagnosed as benign pilomatricoma, leading to a delayed diagnosis; occasionally, it has been misidentified as a vascular malformation. 3 It can be confused between malignant and benign pilomatricoma since both have features of slow progression and firm subcutaneous lesions. 1

Diagnosing malignant pilomatricoma can be challenging due to the lack of distinct histologic criteria that differentiate it from benign pilomatricoma. 2 3 8 Both benign and malignant pilomatricomas exhibit nests of basaloid and eosinophilic, enucleated ghost cells. 1 2 8 However, nuclear polymorphisms, atypical cells, multiple mitoses, and necrosis with serial desmoplasia can aid in distinguishing malignant tumors from their benign counterparts. 1 4 6 Malignant pilomatricoma also presents with poor circumscription; infiltration of the hypodermis, dermis, and cartilage; and occasionally perineural or vascular invasion. 2 This malignancy is characterized by the proliferation of large, anaplastic hyperchromatic basophilic cells with numerous mitoses in various areas, particularly around the tumor's peripheral region, as demonstrated in this case. 4 6 Malignant pilomatricoma can be differentiated from benign tumors by its aggressive features, bony invasion, rare metastasis, and tendency to recur, especially when incompletely excised. 3 4 6 8

The description of histopathology results should encompass the presence of necrosis, atypical mitotic counts, and the occurrence of perineural or vascular invasion. 1 2 Additionally, it is crucial to include the depth of invasion, as poorly differentiated tumors with extensive soft tissue invasion are associated with a poor prognosis. 2 7

No specific immunohistochemical marker for malignant pilomatricoma has yet been confirmed. In this case, the mass was strongly positive for creatine kinase and epithelial membrane antigen. 1 8

Due to the rarity of malignant pilomatricoma, no clear criteria have been established for its surgical management, nor do clear recommendations exist regarding the width of the surgical margin. 8 Based on the limited number of cases reported thus far, a high likelihood of recurrence has been observed when only a simple excision is performed. However, research has confirmed that the recurrence rate is significantly reduced when wide local excision is performed with a safety margin of at least ≥5 mm. 1 2 4 6 9

When developing a treatment plan, it is important to consider the high recurrence rate of malignant pilomatricoma while also noting that distant metastasis is rare. Distant metastases have been reported in approximately 10% of cases, primarily affecting the lungs, bones, regional lymph nodes, and visceral organs, and proving fatal in these instances. 6 7 It can disseminate via the lymphatic or the blood system, and although metastases were initially considered to be exceptional, this contributes to the increase of cases of metastasis. Following surgical treatment, radiotherapy is recommended for cases involving recurrence or residual macroscopic tumors. 1 2 4 However, no established chemotherapy regimen has been demonstrated to be effective in slowing or preventing the progression of malignant pilomatricoma. 7 To the best of our knowledge, systemic chemotherapy has been tried in several cases of malignant pilomatricoma with multiple metastases, and most cases did not respond to treatment. 10 In some cases, however, follow-up chest CT scans showed partial response of metastatic lung lesions after each course of cisplatin and 5-fluorouracil. 11

Malignant pilomatricoma is a rare cutaneous tumor typically found on the head and neck; however, in the present case, it was located on the flank. Diagnosing malignant pilomatricoma can be challenging due to the lack of clear histologic criteria to differentiate it from benign pilomatricoma. For treatment, wide local excision may be sufficient for this carcinoma. Additionally, adjunctive radiotherapy and chemotherapy can be considered in cases of recurrence or metastasis, with the most common locations for metastasis being regional lymph nodes and the lungs.

Malignant pilomatricoma is a rare cutaneous malignancy that is typically found on the head and neck. However, in the case presented herein, it was located on the flank and showed intralesional calcification and multinucleated giant cells. This carcinoma can be treated with wide local excision. Adjunctive radiotherapy can be considered in cases of recurrence or metastasis. The most common locations of metastasis are the regional lymph nodes and the lungs. In the cases with lung metastasis, chemotherapy with cisplatin and 5-fluorouracil can be considered.
